# Protein quality evaluation: FAO perspective

**DOI:** 10.3389/fnut.2024.1446879

**Published:** 2024-12-02

**Authors:** Maria Xipsiti

**Affiliations:** Food and Nutrition Division, Food and Agriculture Organization of the United Nations, Rome, Italy

**Keywords:** amino acids, protein quality, digestibility, PD-CAAS, DIAAS

## Abstract

United Nations agencies have a unique role in achieving the Sustainable Development Goals (SDGs) and aligned global nutrition targets by 2030. According to the latest estimates the world is moving backward in its efforts to end hunger, food insecurity and malnutrition in the presence of a more challenging and uncertain context, including climate change, war conflicts and other challenges. Shifts to plant and novel foods such as insects have been suggested to have good nutritional quality, as well as less environmental impact compared to “traditional” animal source foods. In the context of changing food systems, considering the nutritional quality of foods is essential and accurately assessing protein quality of foods is particularly important, given the large variability in amino acid composition and digestibility between dietary proteins. Indeed, protein quality estimates have the potential to inform policies and programs for actions to improve nutrition throughout the world and have been discussed during past and recent expert consultations. Recently, the Food and Agriculture Organization of the United Nations has been working with the International Atomic Energy Agency and international experts to review and update evidence and related methods on protein quality assessment and to develop a Protein Digestibility Database to aid dialog on the evaluation of protein quality and protein sufficiency in different populations.

## Introduction

With the global population projected to reach 11 billion by the year 2050, sustainably nourishing the world’s population is one of the most pressing challenges we face, which is compounded by the acceleration of climate change (1). Notably, according to the 2023 State of Food Security and Nutrition in the World (SOFI) report, the world is moving backward in its efforts to end hunger, food insecurity and malnutrition (2), with global estimates indicating that among children under 5 years old, around 148.1 million were stunted (22.3 per cent), 45 million were wasted (6.8 per cent) and 37 million were overweight (5.6 per cent) (2). The aftermath of COVID-19 pandemic and the health impacts of climate change, including malnutrition, are increasingly clear especially in the Global South (3). The consequences of malnutrition are enormous, including avoidable ill-health and premature death, as well as significant economic and societal costs (4).

Alongside the wider international system, United Nation Agencies, including the Food and Agriculture Organization of the United Nations (FAO), have a unique role to play promoting lasting solutions to malnutrition as part of a wider sustainable food systems transformation. Better nutrition is one of the pillars of FAO’s Strategic Framework (2022–2031), which articulates FAO’s vision of a sustainable and food secure world for all (5) in the context of the Agenda 2030 for Sustainable Development (6). The right to adequate food established and transition toward healthy diets for national populations is of crucial importance for the enjoyment of all human rights (7) and is at the core of better nutrition, alongside better production, better environment and better life (5). In this regard, providing an adequate, sustainable and nutritious supply of nutrients, including protein, is of critical importance and defining accurately the amount and quality of protein required to meet nutritional needs and describing appropriately the protein supplied by foods and diets is essential. United Nation Agencies, FAO, World Health Organization (WHO) and others have a long history spanning over 50 years in leading the work on establishing global nutrient requirements and coordinating discussions on accurately measuring protein quality in foods and diets.

## Dietary protein

Dietary proteins provide nitrogen (N) and amino acid (AA) and must be supplied by the diet in adequate quantity and proportion. Dietary protein account for a significant part of animal and plant tissues and microorganisms, contributes to metabolism and homeostasis and plays an essential role in human health for growth, maintenance, reproduction, and immune function (or immunity) (8).

The general dietary requirement for protein is defined as an estimated average requirement (EAR) and recommended dietary allowance (RDA). For healthy adults at maintenance, the estimated average requirement for protein based on N balance experiments is 0.66 g/kg body weight/day, and the Recommended Dietary Allowance (RDA) or Population Reference Intake (PRI) is 0.83 g/kg body weight/day (9). Recommendations are also provided for infants and children, and for women during pregnancy and lactation, by including additional components of protein needs by a factorial approach (8). However, protein consumption differs globally and particularly in low-and middle-income countries (LMICs), the amount of protein consumed is consistently lower than in high income countries (HICs), especially for proteins from animal source foods.

At the same time, alternative and novel protein market is growing, with consumers being increasingly exposed to new foods, some of which are novel propositions (plant-based meat alternatives) while others are traditional food items currently introduced to new geographic areas (e.g., tofu and species of edible insects). Cell-based production, which is the field of growing animal agricultural products directly from cell cultures, also continues to expand and has been explored as an alleged sustainable alternative to the conventional livestock agricultural system (10). To ensure the efficacy of novel proteins for widespread consumption, determining their safety through the appropriate regulatory framework is critical. The nutritional value of these protein sources and protein-rich products is subject to variability and depends on their protein content, AA profile and digestibility.

There are two distinct uses of protein quality data: assessment of a diet’s ability to meet human protein and AA requirements, and assessment of the protein adequacy for regulatory purposes of foods and food products sold to consumers (11). How to accurately measure protein quality has been a subject of debate among experts for many years. Proteins are made up of 20 AAs, of which nine are termed indispensable amino acids (IAA), as they are essential but cannot be synthesized in the human body (8). The quality of a protein is defined by its ability to meet age specific nitrogen and IAA requirements for growth, maintenance and specific physiological states (8). Factors affecting the protein quality of a food are the total protein content, the IAA content of the proteins in the food and the metabolic availability of the AAs.

### Regulatory and policy implications of protein quality evaluation

Protein quality estimates are used to inform policies and programs for actions to improve nutrition throughout the world. They are closely tied to food composition data which serve as a critical resource, offering crucial information about the amino acid profiles and digestibility of various protein sources. Various stakeholders, including research institutions, governments and industries with varying levels of expertise, utilize this data to calculate the protein quality of individual foods and mixtures of foods. Additionally, they can be used to evaluate the protein quality of local food sources, guiding agricultural practices to promote the cultivation of high-quality protein crops, thereby improving food security and nutrition.

Regulatory bodies use these estimates to shape international food policies, food security programs and national dietary assessments. Specifically, standardized data on food protein quality in humans can inform recommendations on protein requirements, as well as compositional requirements for foods for special meals. This includes advice on appropriate amino acid complementation or supplementation to enhance the quality of traditional plant-based diets and for setting specialized nutrition standards.

Protein quality guidance also supports the development of food-based dietary guidelines (FBDGs), which provide national recommendations on foods, food groups and dietary patterns for providing required nutrients to the general public to promote overall health and prevent chronic diseases (12). These guidelines are intended to establish a basis for public food and nutrition, health and agricultural policies and nutrition education programs to foster healthy eating habits and lifestyles. They are particularly important in addressing malnutrition in vulnerable populations. Moreover, scientific advice on protein quality evaluation is relevant for the development of Codex Alimentarius food standards and guidelines including information provided on food labels, such as nutrition labeling and protein content claims. The Codex Committee on Nutrition and Foods for Special Dietary Uses (CCNFSDU) has addressed the issue of protein quality in foods and diets on several occasions. In 2019, the FAO and the WHO issued guidance on nitrogen to protein conversion factors for estimating the protein content of soy-based and milk-based ingredients used in infant formulas and follow-up formulas (13) to support the development of the Codex Standard for Follow-up Formula (CXS 156–1987) (14). Additionally, a FAO Expert Working Group provided scientific advice on Protein Quality Assessment in Follow-up Formula for Young Children and Ready to Use Therapeutic Foods (RUTF), outlining future research recommendations for different protein sources (15). This was followed by the provision of FAO supplementary guidance to members of the CCNFSDU uses on computing the Protein Digestibility-Corrected Amino Acid Score (PDCAAS) in follow up formulas (16, 17).

### Protein quality evaluation

For FAO, setting global human nutrient and energy requirements has been an important part of the organization’s work since its founding, with 65 years in setting nutrient requirements, also in collaboration with WHO, and establishing guidelines on diet and nutrition. The determination of protein requirements for human nutrition was reviewed by FAO for the first time in 1955 (18) and in subsequent years with the WHO (8).

Related expert meetings on protein quality evaluation have been discussed over the past decades. In 1989, following a request by the Codex Committee on Vegetable Protein, for determining protein quality in the human diet PDCAAS was adopted by a joint FAO/WHO expert consultation as the most suitable approach for the routine evaluation of overall protein quality for humans and its adoption was recommended as an official method to assess protein quality at international level (19). In calculating PDCAAS the limiting AA score (i.e., the ratio of the first-limiting AA in a gram of target food protein to that in a reference protein or requirement value) is multiplied by protein digestibility, with the intention of assessing how well dietary protein can match the demand for AAs and allowing the prediction of dietary protein utilization (19).

However, the PDCAAS method has received criticism since its adoption. In 2002, the joint FAO/WHO/UNU Expert Consultation on Proteins and Amino Acids in Human Nutrition reviewed the validity of these criticisms recognizing that PDCAAS had several shortcomings (8). In short, PDCAAS does not assign additional nutritional value to proteins with high biological value, it overestimates the nutritional value/protein digestibility of foods that contain antinutrients, and it overestimates the protein digestibility of foods with low digestibility when supplemented with the corresponding limiting AA. The expert consultation recommended that an additional expert consultation be convened to review the validity of PDCAAS for protein quality assessment, suggest appropriate revisions to the method, or adopt a better method applicable to a wider range of human diets (8).

Recognizing limitations in PDCAAS and new research findings, in 2011, FAO convened an expert consultation to review methods for determining dietary protein quality to reflect current best practices (20). A new method for protein quality assessment, the Digestible Indispensable Amino Acid Score (DIAAS,) was proposed as a method for dietary quality assessment for regulatory purposes (20). Experts noted that ileal protein digestibility better reflects the true quantity of AAs digested and absorbed and should be used in calculating DIAAS, as well as that in dietary protein quality evaluation, dietary AA should be treated as individual nutrients and wherever possible digestible or bioavailable AA data should be given in food tables.

However, knowledge and research gaps were also noted, most importantly that there was a lack of human digestibility data available that utilized DIAAS (20). Indeed most existing AA digestibility data came from the pig model, and there was also a lack of public health impact analysis prior to the adoption of DIAAS as the standard method for protein quality assessment. At the time, a move toward DIAAS could have had significant implications for protein requirements and scientific advice for human protein nutrition. Therefore, experts recommended that further research utilizing DIAAS in human subjects was needed before this method could be adopted (20).

Following the 2011 dietary protein quality assessment in human foods, the FAO convened in 2014 an expert working group to update recent advances in protein quality assessment and to discuss the most appropriate methodologies for measuring protein digestibility and utilization in humans (21). The working group aimed to propose and agree on research protocols using both human and animal models to evaluate the ileal AA digestibility of human foods, particularly foods and diets consumed in LMICs (21). Five research protocols in use at the time or that had the potential for further development were recommended for measuring DIAAS, namely: the true ileal digestibility of AA, the use of a dual stable isotope tracer, oxidation of indicator AAs, utilization of postprandial proteins, and net postprandial protein utilization. Experts further recommended to establish a robust database of protein digestibility of foods commonly consumed worldwide, including those consumed in low-income countries along with recommendations to advance research and data collection.

### Toward the development of a database

With funding from the Public Health Agency of Canada, the FAO, in collaboration with IAEA, has initiated the development of a protein quality database. A technical meeting held in October 2022 highlighted the urgent need to create and populate this database, as sufficient data now exists (22). Developing and hosting this database falls within one of FAO’s core functions, to “assemble, analyze, monitor and improve access to data and information, in areas related to FAO’s mandate” (23). The FAO publishes several databases and encourages their use for statistical, scientific, and research purposes, while also offering expertise to guide countries on using the data to help strengthen evidence-based decision-making in the food and agriculture sectors a. Such a data platform is expected to significantly benefit LMICs and smaller nations that lack the technical and financial resources to collect protein quality data and may not otherwise have free access to such information.

Experts at the Joint FAO-IAEA technical meeting (22) presented available and valid models that look at ileal AA digestibility, noting that *in vitro* methods are the way forward, with recommendations for optimization and standardization. They also emphasized the need for collection of additional data to assess the effects of processing, preparation and storage on protein quality, as well as protein quality data from mixed meals, complex foods, and complementary foods. Additionally, they highlighted the need for collection of digestibility data from alternative and novel protein sources, including climate resilient crops, as well as protein quality data from foods. In the discussion on AA requirements and respective reference patterns, members agreed that there are currently no sufficient data to justify setting new requirements, however recommending the need for generating data from vulnerable population groups (with focus on infants and elderly).

Practical steps have been taken to make the database a reality. A FAO/IAEA protein quality database technical advisory group has been established consisting of field experts and secretariat members from the main UN agencies (FAO and IAEA). This group will provide feedback, input and recommendations as needed to guide the construction of the joint FAO-IAEA database and to provide up to date information on the protein quality from food sources, according to the appropriate scoring method. Key actions include formulating and publishing calls for data to populate the database and establishing a framework for its validation that, which allow for data use across various domains. The database will ultimately be populated with peer-reviewed published data and unpublished microdata from these sources, enabling comprehensive meta-analyses to be carried out. A technical advisory group meeting is scheduled for November 2024 to advance the database construction and evaluate the necessary actions for its finalization.

## Discussion

Many individuals do not have access to safe, affordable healthy diets needed to promote health and wellbeing (24) with healthy diets being of reach for more than 3.1 billion people (2). As a result, malnutrition in all its forms is a problem of global proportion, and no country is free from its effects. A healthy diet is one which promotes growth and development and prevents malnutrition. One of the nutrients most discussed in this regard is protein, as there is variability in the contribution of dietary proteins to human nutrient requirements, due to differences in AA composition and in digestibility (25).

An accessible robust database of ileal AA digestibility of individual, complex foods and diets commonly consumed in different parts of the world is needed for informed decisions regarding protein quality using DIAAS. Such a data platform is expected to also benefit LMICs and small countries that lack the technical and financial resources to collect protein quality data and may not otherwise have free access to such data.

The database will be the first of its kind, where comprehensive data on the protein content, AA composition and ileal digestibility of proteins and individual AAs in foods, collected using any accepted validated method (human, pig, rat, *in vitro*), is available free of charge. Data on any food that is part of human diets will be included, covering plant and animal foods and novel protein sources, with a conscious effort to include foods from LMICs, underutilized foods and climate resilient crops. Various processing and food preparation methods and post-harvest storage conditions will be covered, as well as proteins in mixed meals and in complementary foods for young children.

Research institutions, governments, and industry with various levels of skill and background knowledge would be able to use the data to calculate the protein quality of individual foods and mixtures of foods. The data would allow public health professionals to provide guidance on translating requirements into foods consumed, based on the dietary patterns of individuals or population sub-groups. It would also allow assessment of complementarity of protein sources, such as combining different foods that complement one another to provide the IAAs as part of a mixed diet, or in combining such foods in food products like complementary foods; as well as on how poorly digestible proteins can be supplemented with limiting AAs in order to improve the quality of some traditional plant-based diets. Finally, following the eventual regulatory adoption of DIAAS by governments, the data can be used by food regulatory agencies to evaluate food health and nutrition claims by industry.

Moving forward and to further advance the research agenda, there is also a need to identify and stimulate the accrual of funds to support research and generate data and human and technical resources. Research should focus on the generation of protein quality data from various foods and diets in Low-and Middle-Income Countries, as well as data on climate- resilient crops to also address increasing sustainability concerns.

## Data Availability

The presenting findings are from past and recent expert working group meetings and consultations held by FAO and other UN agencies.
